# The Effects of Particulate Matter Sources on Daily Mortality: A Case-Crossover Study of Barcelona, Spain

**DOI:** 10.1289/ehp.1103618

**Published:** 2011-08-16

**Authors:** Bart Ostro, Aurelio Tobias, Xavier Querol, Andrés Alastuey, Fulvio Amato, Jorge Pey, Noemí Pérez, Jordi Sunyer

**Affiliations:** 1 Centre for Research in Environmental Epidemiology, Barcelona, Spain; 2 Institute of Environmental Assessment and Water Research, Spanish Research Council, Barcelona, Spain

**Keywords:** cardiovascular, mortality, particulate matter, PM_2.5_, sources

## Abstract

Background: Dozens of studies link acute exposure to particulate matter (PM) air pollution with premature mortality and morbidity, but questions remain about which species and sources in the vast PM mixture are responsible for the observed health effects. Although a few studies exist on the effects of species and sources in U.S. cities, European cities—which have a higher proportion of diesel engines and denser urban populations—have not been well characterized. Information on the effects of specific sources could aid in targeting pollution control and in articulating the biological mechanisms of PM.

Objectives: Our study examined the effects of various PM sources on daily mortality for 2003 through 2007 in Barcelona, a densely populated city in the northeast corner of Spain.

Methods: Source apportionment for PM ≤ 2.5 μm and ≤ 10 µm in aerodynamic diameter (PM_2.5_ and PM_10_) using positive matrix factorization identified eight different factors. Case-crossover regression analysis was used to estimate the effects of each factor.

Results: Several sources of PM_2.5_, including vehicle exhaust, fuel oil combustion, secondary nitrate/organics, minerals, secondary sulfate/organics, and road dust, had statistically significant associations (*p* < 0.05) with all-cause and cardiovascular mortality. Also, in some cases relative risks for a respective interquartile range increase in concentration were higher for specific sources than for total PM_2.5_ mass.

Conclusions: These results along with those from our multisource models suggest that traffic, sulfate from shipping and long-range transport, and construction dust are important contributors to the adverse health effects linked to PM.

Particulate matter (PM) air pollution is a heterogeneous mix of chemical elements and sources. Although dozens of studies now link exposure to ambient PM with increases in both mortality and morbidity (e.g., Pope and Dockery 2006), considerable uncertainty remains about the relative toxicity of its different sources and constituents. Reports on future research needs for PM ≤ 2.5 μm and ≤ 10 µm in aerodynamic diameter (PM_2.5_ and PM_10_) from both the U.S. National Academy of Science (National Research Council 2004) and the European Commission (2004) stressed identifying the specific components and sources of the PM composition that are most harmful to the exposed population. Epidemiologic studies that examine the health impacts of specific sources of PM, therefore, are critical to addressing this uncertainty. Knowledge of the species and sources of concern would help prioritize research on the biological mechanism for PM effects and help target future pollution control strategies.

In beginning to address this issue, several epidemiologic studies have examined the impact of specific components of PM on both mortality and morbidity (Bell et al. 2009; Franklin et al. 2008; Ostro et al. 2007; Peng et al. 2009; Sarnat et al. 2008; Zanobetti et al. 2009). These studies, which examine the daily association between PM and adverse health over time, lend important insight into the relative toxicity of the myriad constituents of PM. However, many PM constituents are highly correlated, are unmeasured, or, when measured, have many values below detection levels. In addition, the constituents of one PM source, such as vehicle exhaust, will vary greatly from those of other sources, such as residual oil combustion or road dust. Thus, analysis of sources exists as an important complement to the study of specific constituents.

To date, only a few studies have examined the effects of multiple sources of PM_2.5_ or PM_10_ (Laden et al. 2000; Mar et al. 2000; Thurston et al. 2005). Such efforts require source apportionment techniques to determine the share of each element within a given source or factor. Several statistical techniques are available to apportion PM into different source classes (Hopke et al. 2006). As a consequence, the U.S. Environmental Protection Agency sponsored a set of studies to evaluate alternative apportionment methods produced by various investigators. Analysis of these methods indicated that they generally produced similar source categories (Thurston et al. 2005). Further, when the health impacts of exposure to these sources were examined, relatively similar effect estimates were obtained (Mar et al. 2006). This suggests that it is reasonable to use these source estimates in epidemiologic studies to determine their impact on various health outcomes.

Although several studies have been conducted in the United States, few have examined sources in Europe, where the PM composition and exposure patterns are quite different. For example, most major European cities tend to be more densely populated than those in the United States and have a much greater share of mobile sources using diesel fuel. Thus, our study focuses on PM_2.5_ and PM_10_ sources in Barcelona, Spain, a city of 100 km^2^ with approximately 1.6 million people (with 4.5 million in the greater metropolitan area) located in the northeast corner of Spain. Barcelona has one of the highest population densities in the world, at approximately 16,000/km^2^—more than three times that of the other major cities of Spain (Madrid, Valencia, and Seville) and such U.S. cities as Chicago and Philadelphia (Población de España 2010; United Nations Cities Statistics 2010). Also, the relatively scarce precipitation increases the accumulation and resuspension of road and urban dust. In this study, we examined the associations between premature mortality and the various sources of both PM_2.5_ and PM_10_ in Barcelona.

## Materials and Methods

*Mortality and covariate data.* Daily data on mortality for residents of the city of Barcelona who died in the city from 2003 through 2007 were obtained from the Barcelona mortality registry (based on the Catalan mortality registry, Barcelona Public Health Agency, Barcelona, Spain). We examined mortality from all causes (minus accidents and homicides) and cardiovascular disease (codes I00–I99 of the *International Classification of Diseases, 10th Revision* (ICD-10) (World Health Organization 1993). Data on daily temperature and humidity were obtained from the National Meteorological Institute (Madrid, Spain), which maintains a station at the airport, 8 km from the city center.

*Exposure estimates.* We used source estimates developed from an analysis that has been published previously (Amato et al. 2009a). Basically, PM data were collected in Barcelona from 2003 through 2007 at an urban background monitoring station located on the roof (two stories high) of the Institute of Environmental Assessment and Water Research. About 150 m away is a large traffic arterial (Diagonal Avenue), which often experiences > 50,000 vehicles/day. Twenty-four-hour averages of PM_2.5_ and PM_10_ were collected using MCV high-volume (30 m^3^/hr) samplers (MCV, S.A., Barcelona, Spain) approximately every 6 days. PM data collected during clear African dust outbreaks were identified following the methodology described by Perez et al. (2008) and excluded from the analysis in order to differentiate it from the other two mineral sources (urban dust and road dust). After this exclusion plus additional exclusions because of possible contamination and errors in weighing, the frequency of PM species data was approximately every sixth day, with differences in the number of days with PM_2.5_ versus PM_10_ data.

Particles were collected on quartz-fiber filters (15 cm diameter, model QF20, Schleicher and Schuell; Sigma-Aldrich, St. Louis, MO) and analyzed following the procedures described by Querol et al. (2001). Concentrations of total carbon (TC) were determined by elemental analysis; aluminum (Al), calcium (Ca), potassium (K), magnesium (Mg), iron (Fe), titanium (Ti), manganese (Mn), phosphorus (P), sulfur (S), sodium (Na), and 46 trace elements, by inductively coupled plasma (ICP) atomic emissions spectrometry and by ICP mass spectrometry; nitrate (NO_3_^–^) and chloride (Cl^–^), by ion chromatography; and ammonium (NH_4_^+^), by specific electrode.

Ultimately, only 26 chemical species were selected for the source apportionment study, based on the signal-to-noise criterion (Paatero and Hopke 2009) and percentage of data above detection limit: Al, arsenic, Ca, cadmium, Cl^–^, chromium, copper (Cu), Fe, K, Mg, Mn, Na, NH_4_^+^, nickel (Ni), NO_3_^–^, P, lead (Pb), rubidium, S, antimony (Sb), tin (Sn), strontium, TC, Ti, vanadium (V), and zinc (Zn).

In addition to the periodic sampling of PM mass and species, PM_10_ and PM_2.5_ mass were also measured every day using optical counters (versions 1107 and 1108; GRIMM Technologies, Douglasville, GA, USA) corrected by intercomparison with MCV high-volume samplers.

Estimates of source contribution were developed from receptor models based on the mass conservation principle:


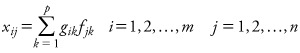
, [1]

where *x_ij_* is the *i*th concentration of the species *j*, *g_ik_* is the *i*th contribution of the source *k*, and *f_jk_* is the concentration of the species *j* in source *k*. When both *g_ik_* and *f_jk_* are unknown, factor analysis techniques such as principal components analysis (Henry and Hidy 1979; Thurston and Spengler 1985) and positive matrix factorization (PMF) (Paatero and Tapper 1994) are used for solving Equation 1. PMF is a weighted least squares method that can be solved using the Multilinear Engine (ME-2) developed by Paatero (1999). ME-2 is a flexible program that permits the incorporation of any *a priori* information such as chemical properties or linear constraints into the model as a target to be fitted to some specified precision. Therefore, ME-2 is especially suitable for source apportionment studies where some knowledge (e.g., chemical ratios, profiles, mass conservation) of involved sources is available and was used for our analysis. Additional details on the technique used have been previously described (Amato et al. 2009a). Identification of factors was also aided by information on their seasonal patterns.

Besides the effects of specific sources, we also examined the effects of total mass concentrations of PM_2.5_ and PM_10_. Our PM mass analysis was performed on two different data sets: a limited data set that included only PM mass measurements for days when species data also were available, and a separate data set that included daily PM measurements during the study period (except for the Saharan dust days), including measurements taken on days when species data were not collected.

*Study design and data analysis.* We used a time-stratified case-crossover study design described by Levy et al. (2001). In this method, the exposure on the date of an event (case) is compared with several nonevent control days (referent periods) occurring on the same month and year. Because all referent periods are selected within the same month as the mortality, seasonal or long-term effects are generally eliminated by design. Variables for temperature, humidity, day of the week, and flu epidemics were also included in the regression model. Temperature and humidity were each modeled using an average of values on the same day of the case (or control) and those of the previous day (i.e., lag 01). We also examined other forms of temperature including 2-, 3-, and 4-day moving averages, quadratic terms, and smoothing splines. Day of week was modeled using six dichotomous variables and was necessary because we did not have enough data to match case and controls by day. A flu epidemic week was designated as a dichotomous variable for a week with incidence rates above baseline levels based on local information (Perez et al. 2008). Each source was then entered separately into the model.

Besides examining the effect of same-day mortality (lag 0), we also considered the effects of exposures on 1–3 previous days (lag 1 to lag 3). However, because data on PM species were not collected every day, a cumulative average could not be investigated. After the basic analysis, we conducted forward stepwise analysis to determine which sources were the best predictors and whether multiple sources were concurrently associated with mortality. We used an inclusion criterion for variable entry of *p* < 0.10. We also created an additional source labeled “traffic” that was the sum of several other sources (described below) and examined this variable in the single- and multisource models. All analyses were conducted using conditional logistic regression in STATA (version 11; StataCorp, College Station, TX, USA). We calculated the excess risk of mortality, defined as (odds ratio – 1) × 100%, and 95% confidence intervals (CIs) for an interquartile range (IQR) increase in each source.

## Results

Eight sources or factors of PM_10_ and PM_2.5_ were identified in the source apportionment model: secondary sulfate/organics (power plants, ship emissions, long-range transport), road dust (brake/tire/road wear and reentrained PM), minerals (urban and construction dust), fuel oil combustion (ship emissions and industrial combustion), industrial (process emissions), secondary nitrate/organics (mobile sources and other fuel combustion), vehicle exhaust, and aged sea salt. Descriptive statistics on the sources, mass concentrations of PM_10_ and PM_2.5_, and mortality are provided in Table 1. For sensitivity analysis, a “traffic” variable was created and set equal to the sum of concentrations of vehicle exhaust (primary PM), road dust, and 70% of the secondary nitrate/organics source. The latter is the approximate share of secondary nitrate/organics due to mobile sources in Barcelona. For PM_2.5_, the mean concentration on days with species data was 26 μg/m^3^, and the dominant sources were vehicle exhaust (30% of the total), secondary sulfate/organics (28%), and secondary nitrate/organics (19%). The average concentration of PM_10_ on days with species data was 42 μg/m^3^, and the dominant sources were minerals (23%), secondary sulfate/organics (18%), vehicle exhaust (18%), and road dust (17%). Mean concentrations of PM_2.5_ and PM_10_ were similar when based on all daily mass samples collected over the 5-year study period. There was an average of 39 and 12 deaths per day from all-cause and cardiovascular mortality, respectively, for both the species-day and every-day analyses.

**Table 1:** Descriptive statistics for mortality and PM mass and sources (µg/m^3^) in Barcelona, 2003–2007.

Variable	Days (*n*)	Daily mean ± SD (IQR)
Mortality				
Total mortality (days with daily PM_2.5_ data)		1,656		38.1 ± 8.8 (10)
Cardiovascular mortality (days with daily PM_2.5_ data)		1,656		12.3 ± 4.3 (6)
Total mortality (days with daily PM_10_ data)		1,725		38.3 ± 8.8 (11)
Cardiovascular mortality (days with daily PM_10_ data)		1,725		12.3 ± 4.3 (6)
Total mortality (days with PM_2.5_ species data)		279		38.1 ± 7.7 (10)
Cardiovascular mortality (days with PM_2.5_ species data)		279		12.3 ± 4.0 (5)
Total mortality (days with PM_10_ species data)		243		38.1 ± 8.0 (10)
Cardiovascular mortality (days with PM_10_ species data)		243		12.1 ± 4.1 (5)
PM mass and sources (μg/m^3^)				
PM_2.5_ mass, species data		279		26.1 ± 11.1 (13.0)
PM_2.5_ mass, daily data		1,656		26.2 ± 11.7 (13.6)
PM_10_ mass, species data		243		41.6 ± 15.7 (20.8)
PM_10_ mass, daily data		1,725		39.6 ± 16.2 (20.6)
PM_2.5_ source				
Secondary sulfate/organics		279		7.3 ± 5.2 (7.4)
Road dust		279		2.3 ± 1.5 (1.8)
Minerals		279		3.2 ± 3.1 (3.1)
Fuel oil combustion		279		1.7 ± 1.5 (1.6)
Industrial		279		0.7 ± 0.6 (0.5)
Secondary nitrate/organics		279		4.9 ± 6.8 (5.5)
Vehicle exhaust		279		7.7 ± 4.2 (5.2)
Aged sea salt		279		0.9 ± 0.9 (0.8)
Traffic		279		13.4 ± 7.4 (9.7)
PM_10_ source				
Secondary sulfate/organics		243		7.3 ± 5.5 (7.5)
Road dust		243		7.0 ± 4.8 (5.9)
Minerals		243		9.6 ± 6.2 (8.2)
Fuel oil combustion		243		2.1 ± 1.6 (1.7)
Industrial		243		0.7 ± 0.7 (0.7)
Secondary nitrate/organics		243		5.1 ± 5.9 (6.5)
Vehicle exhaust		243		7.3 ± 4.3 (5.2)
Aged sea salt		243		3.8 ± 3.1 (4.0)
Traffic		243		17.8 ± 9.1 (11.0)

**Figure 1:**
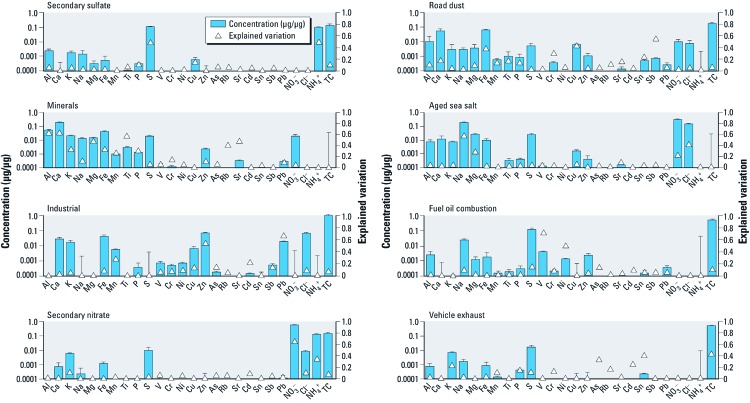
Source profiles (species concentrations within the source) and explained variation (EV) of each species. Error bars represent two times the SD of bootstrap repetitions. EV value indicates the proportional distribution of each species among the different sources (i.e., sum of all EVs for one species among all sources and residuals must equal 1). Abbreviations: As, arsenic; Cd, cadmium; Cr, chromium; Rb, rubidium; Sb, antimony; Sr, strontium.

Figure 1 provides details regarding the estimated source profiles, indicating the concentration of each specific species within each estimated source. For example, for vehicle exhaust, the largest constituents were TC, S, and K, whereas the source identified as minerals consisted primarily of Ca, Al, and Fe, among the analyzed species. Figure 1 also provides information about the explained variation (EV). As described by Paatero (2000), the EV indicates the importance of each factor in explaining the variation of a given species. It measures the contribution of each source to the ambient air concentrations of each chemical species and can therefore be useful for qualitative identification of the sources. For example, a factor that explains a great proportion (i.e., high EV values) of V and Ni would be identified as a fuel oil combustion emission source. Likewise, Figure 1 shows that the industrial factor explains most of the variation (high EV values) for Mn, Pb, and Zn.

Table 2 summarizes correlations among the estimated sources. For both PM_2.5_ and PM_10_, correlations were fairly modest, generally with 0.1 < *r* < 0.4. Among the highest correlations for PM_2.5_ were vehicle exhaust with road dust (0.39) and secondary sulfate (–0.40).

**Table 2:** Correlation among the estimated sources of PM_2.5_ and PM_10_.

Particle size and source	SS	RD	MI	FO	IN	SN	VE	AS
PM_2.5_																
Secondary sulfate/organics		1														
Road dust		–0.07		1												
Minerals		0.02		0.20		1										
Fuel oil combustion		0.30		0.14		0.07		1								
Industrial		0.21		0.20		0.08		0.04		1						
Secondary nitrate/organics		0.16		0.09		–0.11		0.16		0.37		1				
Vehicle exhaust		–0.40		0.39		0.04		–0.13		0.12		0.16		1		
Aged sea salt		0.08		0.00		0.07		–0.07		–0.06		–0.22		–0.27		1
PM_10_																
Secondary sulfate/organics		1														
Road dust		–0.11		1												
Minerals		0.06		0.13		1										
Fuel oil combustion		0.26		0.24		0.23		1								
Industrial		0.24		0.19		0.20		0.16		1						
Secondary nitrate/organics		0.28		0.36		0.06		0.33		0.33		1				
Vehicle exhaust		–0.34		0.36		0.24		–0.15		0.00		–0.12		1		
Aged sea salt		–0.02		–0.36		–0.07		–0.12		–0.19		–0.27		–0.13		1
Abbreviations: AS, aged sea salt; FO, fuel oil combustion; IN, industrial; MI, minerals; RD, road dust; SN, secondary nitrates/organics; SS, secondary sulfates/organics; VE, vehicle exhaust.																

**Figure 2:**
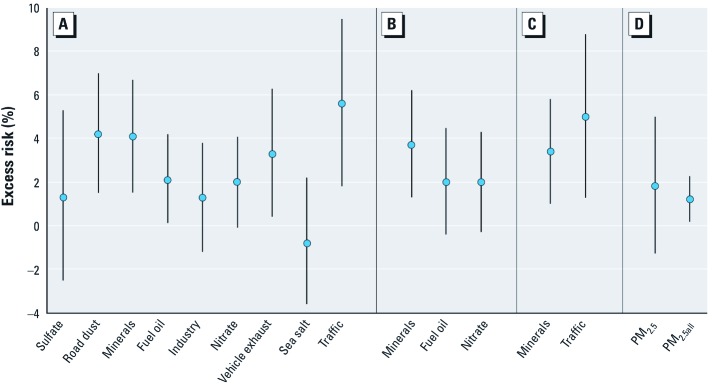
All-cause mortality excess risks (95% CIs) associated with IQR increases in sources of PM_2.5_ (lag 2): single-source models (*A*), multisource models (*B*), multisource models with traffic (*C*), and PM mass models (*D*). PM_2.5_, mass from periodic sampling; PM_2.5all_, mass from daily sampling.

Figure 2 summarizes the regression results for all-cause mortality and sources in PM_2.5_ for a 2-day lag, because the model fit was best for this lag. A full set of results for all lags is provided in the Supplemental Material, Table 1 (http://dx.doi.org/10.1289/ehp.1103618). Based on single-source models, statistically significant associations (*p* < 0.05) were observed between mortality and road dust, minerals, fuel oil combustion, and vehicle exhaust with a 2-day lag. In general, we observed excess risks of around 2–4% as central estimates for a change in the respective IQRs of each of the eight original estimated sources. In contrast, for the composite traffic variable the excess risk was almost 6% for an IQR change. For PM_10_, significant associations were observed only for minerals, vehicle exhaust, and traffic, with excess risks that were fairly similar to those produced from the sources of PM_2.5_ [see Supplemental Material, Table 1 (http://dx.doi.org/10.1289/ehp.1103618)]. The source results were unchanged when we used other temperature metrics (i.e., moving averages, smoothing splines) in the regression models (data not shown).

For the stepwise regression of the eight PM_2.5_ sources, a 2-day lag was used for each source because this was the lag that best predicted mortality. Three PM_2.5_ sources met the stepwise regression inclusion criteria of *p* < 0.10: minerals, fuel oil combustion, and secondary nitrate/organics (Figure 2B). The resultant excess risks were generally similar to those generated when the sources were entered separately into the model. When we performed a stepwise regression that included a factor that encompassed the full effects of traffic, two factors met the inclusion criterion, minerals and traffic, with excess risks of 3% and 5%, for their IQRs, respectively (Figure 2C). Because few PM_10_ sources were associated with mortality, a multisource model was not examined for this pollutant.

Significant associations with all-cause mortality were not seen for PM_2.5_ and PM_10_ based on the limited (i.e., approximately every sixth day) data set that was restricted to the days when species data were collected, but significant associations were observed based on the full, daily data set of approximately 1,700 observations. Lags of 0, 1, and 2 days were all associated with mortality [Figure 2D; see also Supplemental Material, Table 1 (http://dx.doi.org/10.1289/ehp.1103618)]. The excess risk for all-cause mortality using a 1-day lag was 1.9% (95% CI: 0.8, 3.1) for an IQR increase in PM_2.5_ of 13.6 μg/m^3^, which corresponds to an excess risk of 1.4% (95% CI: 0.6, 2.3) for a 10-μg/m^3^ increase in PM_2.5_. The excess risk of all-cause mortality for the IQR for PM_10_ of 20.6 μg/m^3^ was 2.4%, or 1.2% (95% CI: 0.6, 1.7) for a 10-μg/m^3^ change [Supplemental Material, Table 1 (http://dx.doi.org/10.1289/ehp.1103618)].

**Figure 3:**
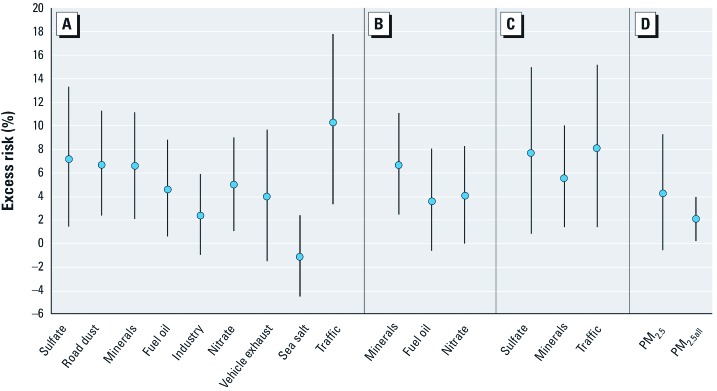
Cardiovascular mortality excess risks (95% CIs) associated with IQR increases in sources of PM_2.5_ (lag 2): single-source models (*A*), multisource models (*B*), multisource models with traffic (*C*), and PM mass models (*D*). PM_2.5_, mass from periodic sampling; PM_2.5all_, mass from daily sampling.

A fairly similar pattern of associations emerged when examining cardiovascular mortality, with a few differences [Figure 3; see also Supplemental Material, Table 2 (http://dx.doi.org/10.1289/ehp.1103618)]. Specifically, statistically significant associations with PM_2.5_ at lag 2 were observed for secondary sulfate/organics but not for vehicle exhaust. As with all-cause mortality, significant associations were also observed for the sources road dust, minerals, fuel oil combustion, secondary nitrate/organics, and traffic (Figure 3). For PM_10_, associations were again observed for minerals, vehicle exhaust, and traffic (see Supplemental Material, Table 2). As expected, the excess risks for cardiovascular mortality were generally higher than those observed for all-cause mortality: 5–7% for the associated IQRs of the eight original PM_2.5_ sources and 10% for the PM_2.5_ traffic factor (Figure 3A). In the multisource model for PM_2.5_, the same factors that were included in the all-cause mortality model met the inclusion criterion for cardiovascular mortality: minerals, secondary nitrate/organics, and fuel oil combustion (Figure 3B). When the “traffic” factor was added to the stepwise regression, it was included in the final model along with minerals and secondary sulfate (Figure 3C). Again, a multisource model for PM_10_ was not examined because few sources were associated with cardiovascular mortality.

Regarding the effects of PM mass on cardiovascular mortality, in the limited species data set, associations were observed for lag 1 PM_2.5_ and PM_10_. For the daily data set, associations with lags of 0, 1, and 2 days were observed for both PM_2.5_ and PM_10_. The excess risk for cardiovascular mortality using a 1-day lag was 3.9% (95% CI: 1.9, 6.0) for an IQR increase in PM_2.5_ of 13.6 μg/m^3^, which corresponds to an excess risk of 2.9% (95% CI: 1.4, 4.4) for a 10-μg/m^3^ increase in PM_2.5_. The excess risk of cardiovascular mortality for the IQR for PM_10_ of 20.6 μg/m^3^ was 5.7%, or 2.8% (95% CI: 1.7, 3.8) for a 10-μg/m^3^ change [see Supplemental Material, Table 2 (http://dx.doi.org/10.1289/ehp.1103618)].

## Discussion

In our analysis of short-term exposure to the estimated sources of PM_2.5_ and PM_10_, we observed several important associations with both all-cause and cardiovascular mortality. Specifically, for all-cause mortality and PM_2.5_, there were significant associations with estimated sources identified as road dust, minerals, fuel oil combustion, vehicle exhaust, and traffic. For cardiovascular mortality, associations were also observed for secondary sulfate/organics. In multisource models, both traffic and mineral sources were significantly associated with all-cause mortality, whereas traffic, minerals, and sulfate were significantly associated with cardiovascular mortality.

For the limited data set where mass was collected concurrently on the days of speciation collection (*n* = 279), no association was observed between PM_2.5_ mass and all-cause mortality. This indicates the importance of the specific sources (and species) because, in contrast, several of them were associated with mortality. For the full 5-year period of daily PM_2.5_ measurements (*n* = 1,656), which also included days when chemical speciation data were not collected, lags of 0, 1, and 2 days were associated with both all-cause and cardiovascular mortality. For this full sample of PM_2.5_ data, the estimated excess risk of mortality per 10 μg/m^3^ (lag 1) was 1.4% (95% CI: 0.6, 2.3), which is within the upper range of those reported in previous multicity studies in the United States (Franklin et al. 2007; Ostro et al. 2006; Schwartz et al. 1996; Zanobetti and Schwartz 2009).

For the analysis using daily PM_2.5_, a 1-day lag provided a slightly better fit, based on *t*-statistics, than either lag 0 or lag 2. With the less frequent PM_2.5_ data collected every 3–6 days, lag 2 provided the best fit. This difference in results for lags is likely due to chance because with the periodic data set, each lag corresponds to a different mortality day. That is, with PM collected on day *t* or PM(*t*), lag 0 corresponds to mortality on day *t*, or *M*(*t*). A 1-day lag relates PM(*t*) to *M*(*t* + 1), whereas a 2-day lag relates PM(*t*) to *M*(*t* + 2). Thus, lags in the source data set correspond to nonoverlapping mortality data sets.

In contrast to PM_2.5_, fewer sources of PM_10_ were significantly associated with mortality. However, significant associations with all-cause and cardiovascular mortality were detected for the sources identified as minerals and vehicle exhaust. For the full sample of days with PM_10_ data, associations were also observed between it and both all-cause and cardiovascular mortality.

In considering the eight single-source categories of PM_2.5_, the highest effect estimates for all-cause mortality with excess risks of 4% per their respective IQRs were observed for the sources vehicle exhaust, road dust, and minerals. The vehicle exhaust particles represent primary emissions from vehicle engines, because secondary inorganic species were not included in this source. Although dominated by TC, this factor also includes S, Fe, Cu, and Sn (Amato et al. 2009a). The estimated mortality effect of short-term exposure to this source amounts to approximately 8% per 10 μg/m^3^ (lag 1), much larger than the estimated 3.4% effect of traffic reported by Laden et al. (2000) for six eastern U.S. cities and the 4.2% effect of traffic for Washington, DC (Thurston et al. 2005). The larger estimated effect for Barcelona may be attributable to greater population density and subsequent exposure to traffic; differences in the mobile source mix, especially the high proportion of diesel vehicles; and/or the source apportionment method itself. When the traffic factor was created and included in the model, it had the smallest *p*-value and largest effect estimate for both all-cause and cardiovascular mortality. This provides additional evidence of the importance of mobile sources.

The second estimated factor, identified as road dust, has significant shares of TC, Fe, Cu, and Sb and likely reflects both brake wear and reentrained particles (Amato et al. 2009a, 2009b). The heavy metals may serve simply as general markers for this factor or may have independent toxic properties of their own. The importance of this factor in Barcelona may be especially attributable to the generally infrequent rainfall, resulting in significant reentrainment of PM in the streets, concurrent with subsequent high exposures for the relatively dense urban population. Several toxicologic studies of PM components have documented the role of these transition metals in enhancing oxidative stress and increasing the production of reactive oxygen species (Maciejczyk et al. 2010; Sangani et al. 2010). Such effects are likely to play a significant mechanistic role in the pathology of air pollution (Kelly 2003).

The third estimated major factor, identified as “minerals,” contains large shares of Ca, Al, and Fe. A previous analysis suggested several sources for this factor, including urban dust, such as construction dust and reentrained particles from unpaved areas, including parking lots and gardens (Amato et al. 2009a). Thus, toxicity might result both from the transition metals described above and from other anthropogenic mineral materials. Days with African dust outbreaks were deleted from the data. However, previous studies have documented evidence of mortality effects in Barcelona from coarse Saharan dust (Perez et al. 2008), and it is likely that some of these particles will be deposited and resuspended even a few days after dust outbreaks, thereby likely affecting the PM concentrations in the PM_10_ and PM_2.5_ size ranges.

Besides these three factors, fuel oil combustion and secondary nitrate/organics were also associated with all-cause mortality, and secondary sulfate was associated with cardiovascular mortality. Sulfate was also one of the three factors that met the inclusion criteria for the multipollutant model of cardiovascular mortality. Based on the analysis of Amato et al. (2009a), the fuel oil combustion factor, which accounts for most of the EV of V and Ni but is mostly made up of TC and S, likely reflects industrial combustion sources but mostly shipping emissions. Their analysis also attributed secondary nitrate/organics mostly to road traffic, and the secondary sulfate factor, to photochemical oxidation of sulfur oxides emitted mostly regionally but also from a few local utilities, shipping, and long-range transport. Although mean levels of sulfur dioxide in Barcelona are relatively low [i.e., in the lowest range of European cities, at ~ 3 μg/m^3^, which is similar to Stockholm’s urban background (Reche et al. 2010)], previous analysis noted a clear correlation with midday sulfate nucleation processes and nanoparticle pollution episodes because of the importance of photochemical nucleation (Reche et al. 2010).

The present analysis suggests an excess risk of about 10% per 10-μg/m^3^ (lag 2) change in sulfate. Several previous studies have reported lower estimated effects on all-cause mortality from short-term exposure to sulfate per 10 μg/m^3^: 2.8% for six U.S. cities (Laden et al. 2000), 3.8% for Washington, DC (Thurston et al. 2005), and 4.8% for Boston (Maynard et al. 2007).

Among the sources of PM_10_, only minerals and vehicle exhaust were significantly associated with either all-cause or cardiovascular mortality. Of note, approximately 75% of the mineral source is in the coarse PM size range, between 2.5 and 10 μm in diameter. As discussed earlier, the mineral factor, dominated by Ca, Al, and Fe, likely reflects urban mineral dust other than road dust. Several previous studies have documented an association between coarse PM and all-cause and cardiovascular mortality in locations such as Palm Springs, California; multiple counties in California; Phoenix, Arizona; and Mexico City (Castillejos et al. 2000; Malig and Ostro 2009; Mar et al. 2000; Ostro et al. 2003). Although PM_10_ from road dust was generally positively associated with mortality, the estimates were not statistically significant.

Taken together, our results suggest that several sources of PM_2.5_ are likely important contributors to adverse health outcomes in Barcelona. This includes PM emanating from mobile sources either directly (vehicle exhaust, secondary nitrate/organics) or indirectly through reentrainment of road dust, shipping and stationary source emissions (fuel oil combustion, secondary sulfate), and mineral dust. Some additional evidence provided by the results of the multisource regression serves to narrow the list to two or three main sources in the final model: traffic (including both primary and secondary PM, and road dust), sulfate and urban dust from construction and demolition. Previous studies have observed associations with multiple sources or tracers of sources. For example, Laden et al. (2000) found evidence of effects on mortality from both motor vehicle exhaust and coal combustion predominantly in U.S. East Coast cities. Ostro et al. (2007) also observed associations between mortality both traffic and biomass for multiple cities in California. Finally, Zhou et al. (2011) found evidence of traffic effects for the warm season in Detroit, Michigan, and the cold season in Seattle, Washington, along with effects from biomass combustion, residual oil, and metals processing in Seattle.

As always, there are some caveats to the interpretation of results. First, the identification of specific sources or factors may be dependent on the analytic methods used. However, results from other epidemiologic studies suggest that the associations are consistent regardless of the methods employed to identify PM sources. For example, studies undertaken in Washington, DC, Phoenix, Arizona, and Atlanta, Georgia, all considered multiple source-apportionment methods for source identification. In these studies, the subsequent analysis of health effects associated with these factors generated fairly similar results (Sarnat et al. 2008; Thurston et al. 2005). In the present study we used a hybrid factor analysis—chemical mass balance source apportionment approach to accurately characterize and subsequently quantify road dust, in addition to other common urban sources.

Because of the relative sparseness of the species data, we fitted the same source profiles for both PM_2.5_ and PM_10_. Performing PMF analysis for PM_10_ and PM_2.5_ separately for this data set resulted in less precise results (Amato et al. 2009a). Given the small number of species data points, therefore, PM data from different size fractions were assembled into a two-dimensional array and analyzed together to significantly increase the number of observations. These combined data displayed the most realistic results for factors profiles. Thus, the variability of factors profiles among different PM sizes could not be investigated. The resulting source profiles and the EV of the species are therefore the same for both PM_10_ and PM_2.5_.

Another caveat is that the sources that were observed to be associated with mortality may be proxy markers of exposure for unmeasured elements or sources that are the underlying causes of the associations. Third, we relied on a single monitor for our estimates of exposure. In general, the resultant biases caused by misclassification of exposure should be toward the null. However, to the extent that the different sources have different spatial exposure patterns, there may be differential misclassification, which could lead to biased results. Finally, it is possible that the results were obtained purely by chance.

## Conclusion

Our study suggests the likelihood of significant health effects in Barcelona resulting from exposure to PM_2.5_ and more specifically from exposure to mobile sources (both exhaust and road dust emissions). There also is evidence that exposure to other sources of PM_2.5_ including reentrained PM, sulfate from shipping and long-range transport, and construction dust contributes to adverse health as well. Thus, our results lend additional support to efforts to control multiple sources of PM_2.5_.

## Supplemental Material

(82 KB) PDF
