# Supplementary material for: The Effects of Particulate Matter Sources on Daily Mortality: A Case-Crossover Study of Barcelona, Spain
Source: Environ Health Perspect. 2011 Aug 16;119(12):1781–7. doi: 10.1289/ehp.1103618 (PMC3261985; doi:10.1289/ehp.1103618)
Supplement: (82 KB) PDF [file ehp.1103618.s001.pdf]

Supplemental Material

The Effects of Particulate Matter Sources on Daily Mortality: A Case-Crossover Study of Barcelona, Spain

Bart Ostro, Aurelio Tobias, Xavier Querol, Andrés Alastuey, Fulvio Amato, Jorge Pey, Noemí Pérez, Jordi Sunyer

Source		PM2.5				PM10			
	Lag	IQR	OR	95% CI		IQR	OR	95% CI	
Secondary Sulfate/Organics	0	7.4	-0.5	-4.8	4.1	7.5	-1.3	-4.9	2.4
	1		-1.5	-5.1	2.2		-1.5	-5.9	3.1
	2		1.3	-2.5	5.3		-0.5	-4.3	3.4
	3		1.9	-2.5	6.5		-0.6	-4.4	3.4
Road Dust	0	1.8	-1.0	-4.9	3.0	5.9	-1.2	-5.8	3.6
	1		2.0	-2.4	6.7		3.0	-2.0	8.2
	2		**4.2**	**1.5**	**7.0**		1.7	-2.5	6.0
	3		1.2	-2.5	5.1		2.8	-0.6	6.3
Mineral	0	3.1	2.1	-0.3	4.6	8.2	0.1	-3.8	4.1
	1		1.5	-0.7	3.7		**4.8**	**1.3**	**8.4**
	2		**4.1**	**1.5**	**6.7**		**5.3**	**0.3**	**10.7**
	3		0.8	-1.4	3.0		2.1	-2.3	6.7
Fuel Oil Combustion	0	1.6	0.0	-3.4	3.5	1.7	-0.3	-3.5	3.1
	1		-1.1	-3.2	1.0		-0.1	-2.7	2.6
	2		**2.1**	**0.1**	**4.2**		1.1	-1.3	3.5
	3		-2.0	-4.0	0.1		-1.4	-3.6	0.9
Industrial	0	0.5	0.3	-1.6	2.1	0.7	-0.2	-2.2	1.8
	1		-0.1	-2.2	2.1		0.6	-1.9	3.1
	2		1.3	-1.2	3.8		0.1	-2.6	2.9
	3		-0.4	-2.9	2.2		-0.8	-3.5	2.0
Secondary Nitrate/ Organics	0	5.5	-1.1	-3.3	1.2	6.5	-3.2	-6.6	0.5
	1		0.2	-2.9	3.5		0.1	-4.4	4.9
	2		2.0	-0.1	4.1		1.9	-1.7	5.6
	3		1.0	-1.8	3.9		1.5	-1.9	5.1
Vehicle Exhaust	0	5.2	-1.1	-4.1	2.0	5.2	1.7	-1.2	4.8
	1		**3.7**	**0.7**	**6.7**		**2.9**	**0.2**	**5.7**
	2		**3.3**	**0.4**	**6.3**		**3.6**	**0.1**	**7.2**
	3		-1.6	-4.7	1.6		-1.0	-3.7	1.8
Aged Sea Salt	0	0.8	0.2	-2.0	2.4	4.0	-0.1	-4.9	5.0
	1		0.3	-2.6	3.3		-0.3	-4.6	4.2
	2		-0.8	-3.6	2.2		0.5	-3.3	4.4
	3		-1.9	-4.0	0.3		-3.0	-6.2	0.3
Traffic	0	9.7	-2.2	-5.4	1.2	11.0	-1.3	-5.3	2.8
	1		3.5	-2.4	9.6		3.3	-1.8	8.7
	2		**5.6**	**1.8**	**9.5**		**3.9**	**0.0**	**8.1**
	3		0.4	-4.0	5.0		1.6	-1.7	5.1
Multi-source:									
Mineral	2	3.1	**3.7**	**1.3**	**6.2**				
Fuel Oil	2	1.6	2.0	-0.4	4.5				
Secondary Nitrate	2	5.5	2.0	-0.3	4.3				
Multi-source#									
Mineral	2	3.1	**3.4**	**1.0**	**5.8**				
Traffic	2	9.7	**5.0**	**1.3**	**8.8**				
PM2.5/PM10 from species data set	0	13.0	0.9	-3.0	5.0	20.8	-0.5	-4.5	3.7
	1		1.2	-2.8	5.4		4.0	-0.2	8.4
	2		1.8	-1.3	5.0		3.6	-1.0	8.3
	3		0.5	-3.4	4.6		0.5	-3.8	5.0
PM2.5/PM10 every day	0	13.6	**1.5**	**0.4**	**2.7**	20.6	**1.5**	**0.3**	**2.7**
	1		**1.9**	**0.8**	**3.1**		**2.4**	**1.2**	**3.6**
	2		**1.2**	**0.2**	**2.3**		**1.8**	**0.6**	**2.9**
	3		0.1	-0.9	1.1		0.7	-0.4	1.8

Table 1. Excess risk for all-cause mortality (Central estimate and 95% Confidence Interval (CI) for interquartile range (IQR)); Bold = statistically significant at $p < 0.05$); #Stepwise regression model included traffic for potential selection

Source	Lag	PM2.5 IQR	PM2.5 OR	PM2.5 95% CI		PM10 IQR	PM10 OR	PM10 95% CI	
Secondary Sulfate/Organics	0	7.4	-0.9	-7.4	6.1	7.5	-1.3	-7.3	5.0
	1		5.6	-1.2	12.9		5.3	-1.4	12.4
	2		**7.2**	**1.4**	**13.3**		2.9	-3.3	9.4
	3		1.7	-4.8	8.7		-2.5	-9.6	5.3
Road Dust	0	1.8	-4.5	-11.0	2.5	5.9	-3.9	-11.4	4.2
	1		0.5	-6.7	8.3		3.5	-5.1	12.8
	2		**6.7**	**2.4**	**11.3**		3.5	-3.4	11.0
	3		0.0	-5.9	6.2		-0.6	-6.8	6.0
Mineral	0	3.1	**5.2**	**1.1**	**9.4**	8.2	0.8	-5.8	7.8
	1		2.7	-0.8	6.3		**5.7**	**0.3**	**11.3**
	2		**6.6**	**2.1**	**11.2**		6.4	-1.7	15.3
	3		1.6	-2.4	5.6		-0.1	-7.2	7.6
Fuel Oil Combustion	0	1.6	-4.4	-9.2	0.7	1.7	-2.8	-7.8	2.5
	1		0.6	-3.0	4.4		0.4	-3.0	4.0
	2		**4.6**	**0.6**	**8.8**		1.9	-2.1	6.1
	3		-0.6	-4.6	3.5		-0.2	-4.4	4.2
Industrial	0	0.5	2.3	-2.0	6.8	0.7	0.9	-2.7	4.6
	1		2.2	-1.3	5.8		2.6	-0.9	6.2
	2		2.4	-0.9	5.9		-0.6	-4.5	3.5
	3		-2.0	-6.0	2.2		-4.2	-8.7	0.6
Secondary Nitrate/ Organics	0	5.5	-1.4	-4.8	2.2	6.5	-1.5	-6.9	4.3
	1		-0.3	-4.6	4.1		-1.1	-7.4	5.6
	2		**5.0**	**1.1**	**9.0**		5.1	-0.9	11.5
	3		0.7	-3.2	4.7		-0.4	-6.4	6.0
Vehicle Exhaust	0	5.2	-2.9	-7.9	2.3	5.2	3.6	-2.3	9.9
	1		2.8	-2.4	8.3		**6.4**	**1.5**	**11.6**
	2		4.0	-1.5	9.7		4.3	-1.0	9.9
	3		-1.9	-6.3	2.7		-4.9	-9.3	-0.3
Aged Sea Salt	0	0.8	1.1	-2.2	4.6	4.0	-1.7	-8.5	5.6
	1		0.7	-2.3	3.7		-1.0	-7.8	6.3
	2		-1.1	-4.5	2.4		4.4	-2.4	11.6
	3		-2.9	-6.2	0.6		-2.3	-8.1	3.9
Traffic	0	9.7	-4.6	-10.7	1.9	11.0	-0.6	-7.8	7.1
	1		1.8	-5.7	10.0		5.0	-3.3	14.0
	2		**10.3**	**3.3**	**17.8**		**7.2**	**0.5**	**14.3**
	3		-0.5	-5.6	4.8		-3.4	-9.0	2.5
Multi-source:									
Mineral	2	3.1	**6.7**	**2.5**	**11.1**				
Fuel Oil Comb	2	1.6	3.6	-0.6	8.1				
Secondary Nitrate	2	5.5	**4.1**	**0.1**	**8.3**				
Multi-source#									
Secondary Sulfate	2	7.4	**7.7**	**0.8**	**15.0**				
Mineral	2	3.1	**5.6**	**1.4**	**10.0**				
Traffic	2	9.7	**8.1**	**1.4**	**15.2**				
PM2.5/PM10 from species data set	0	13.0	-0.3	-6.3	6.1	20.8	-2.4	-9.5	5.2
	1		**7.3**	**0.7**	**14.2**		**10.0**	**3.0**	**17.5**
	2		4.3	-0.5	9.3		5.3	-2.6	13.8
	3		2.5	-3.5	8.8		-2.0	-9.2	5.7
PM2.5/PM10 every day	0	13.6	**2.4**	**0.4**	**4.4**	20.6	**2.0**	**0.0**	**4.1**
	1		**3.9**	**1.9**	**6.0**		**5.7**	**3.5**	**7.8**
	2		**2.1**	**0.2**	**4.0**		**3.5**	**1.5**	**5.6**
	3		0.1	-1.7	1.9		0.7	-1.3	2.7

Table 2. Excess risk for cardiovascular mortality (Central estimate and 95% Confidence Interval (CI) for interquartile range (IQR)); Bold = statistically significant at $p < 0.05$); #Stepwise regression model included traffic for potential selection
